# Significant salivary changes in relation to oral mucositis following autologous hematopoietic stem cell transplantation

**DOI:** 10.1038/s41409-020-01185-7

**Published:** 2021-01-08

**Authors:** S. J. M. van Leeuwen, G. B. Proctor, A. M. G. A. Laheij, C. M. J. Potting, O. Smits, E. M. Bronkhorst, M. D. Hazenberg, T. M. Haverman, M. T. Brennan, I. von Bültzingslöwen, J. E. Raber-Durlacher, M. C. D. N. J. M. Huysmans, F. R. Rozema, N. M. A. Blijlevens

**Affiliations:** 1 Radboud Institute for Health Sciences, Department of Dentistry, Radboud University Medical Center, Nijmegen, The Netherlands; 2 Centre for Host Microbiome Interactions, Faculty of Dentistry, Oral & Craniofacial Sciences, King’s College, London, UK; 3 Department of Oral Medicine, Academic Centre for Dentistry Amsterdam, University of Amsterdam and VU University, Amsterdam, The Netherlands; 4 Department of Preventive Dentistry, Academic Centre for Dentistry Amsterdam, University of Amsterdam and VU University, Amsterdam, The Netherlands; 5 Radboud Institute for Health Sciences, Department of Hematology, Radboud University Medical Center, Nijmegen, The Netherlands; 6 Department of Hematology, Amsterdam UMC, University of Amsterdam, Amsterdam, The Netherlands; 7 Department of Oral Medicine, Atrium Health’s Carolinas Medical Centre, Charlotte, NC USA; 8 Department of Oral Microbiology and Immunology, Institute of Odontology, The Sahlgrenska Academy, University of Gothenburg, Gothenburg, Sweden; 9 Department of Oral and Maxillofacial Surgery, Amsterdam UMC, University of Amsterdam, Amsterdam, The Netherlands

**Keywords:** Myeloma, Biochemistry, Bone marrow transplantation

## Abstract

The aim of this multicentre, longitudinal study was to determine salivary changes in relation to oral mucositis (OM) in multiple myeloma patients following high-dose melphalan and autologous hematopoietic stem cell transplantation (ASCT). Unstimulated and stimulated whole-mouth saliva samples (UWS and SWS) were collected before ASCT, 1×/wk during the hospitalisation phase, and 3 and 12 months post-ASCT. During the hospitalisation period OM was scored 3×/wk (WHO system). Flow rate, pH, total protein concentration (Nanodrop), albumin, lactoferrin, neutrophil defensin-1 (HNP1), total IgA and S100A8/A9 (ELISA) were determined. Mixed models were used to evaluate differences between ulcerative (u)OM (≥2 WHO, *n* = 20) and non-uOM (*n* = 31) groups. Until 18 days after ASCT, flow rate, pH, total IgA and HNP1 levels decreased in UWS and/or SWS, while log lactoferrin levels were significantly increased (UWS: *p* = 0.016 95% CI [0.36, 3.58], SWS: *p* < 0.001 95% CI [1.14, 3.29]). Twelve months post-ASCT, salivary protein levels were similar to baseline except for log total IgA, which was higher (UWS: *p* < 0.001 95% CI [0.49, 1.29], SWS: *p* < 0.001 95% CI [0.72, 1.45]). No differences between uOM and non-uOM groups were observed. Changes in salivary proteins indicated an inflammatory reaction in salivary glands coinciding with mucosal and systemic reactions in response to high-dose melphalan.

## Introduction

A common and most debilitating complication of high-dose melphalan (HDM) before autologous hematopoietic stem cell transplantation (ASCT) is oral mucositis (OM), clinically characterised by erythema, oedema and ulcerations [1, 2]. Patients’ symptoms include pain and a dry mouth [2]. Although local vasoconstriction by ice chips (cryotherapy) during the infusion of melphalan significantly reduces the incidence of OM, it is still present in up to 44% of patients [3].

Saliva, a mixture of water, ions and proteins, is mainly produced by three pairs of major salivary glands: the parotid, submandibular and sublingual glands. The contribution of each gland is different between unstimulated and stimulated saliva. In stimulated saliva, the parotid gland is more active resulting in a more watery saliva [4]. Saliva has several functions including lubrication and protection, mainly related to proteins [4]. Mucins play a major role in lubrication, while lactoferrin, defensins and IgA have antimicrobial activities and are important for the protective function [5].

IgA is the main immunoglobulin found in saliva, and it is responsible for the humoral immune response at the oral mucosa [6]. Binding of (secretory) IgA limits mucosal colonisation and subsequent invasion of microorganisms [6]. Polymorphonuclear leucocytes (PMNs) and oral keratinocytes also play a role in the innate immunity of the oral mucosa [6, 7]. Since the azurophilic granules of PMNs are the only source of neutrophil defensin-1 (HNP1) [8], it could be used as a marker of PMNs in saliva. Another product in the granules of PMNs and oral keratinocytes found in saliva is S100A8/A9 (or calprotectin) [9, 10].

As a consequence of the chemotherapy and/or radiotherapy before an autologous and allogeneic SCT, changes in salivary flow rate and salivary proteins have been reported [11]. Decreasing trends were reported for salivary flow rate and salivary IgA levels up to 1 month after autologous and allogeneic SCT, while albumin and lactoferrin levels were increased in the first weeks and up to 1 and 6 months, respectively, after autologous and allogeneic SCT [11–14].

A low salivary flow rate was identified as a risk factor for OM in patients receiving the chemotherapeutic agent 5-fluorouracil [15]. Whether changes in salivary proteins are associated with OM and to what extent is still unknown. Therefore, the aim of this longitudinal, multicentre study was to determine salivary changes in relation to OM in multiple myeloma (MM) patients following HDM and ASCT.

## Materials and methods

### Study population

This study was part of the Ora-stem study, an international, prospective, longitudinal, multicentre study to investigate oral problems after conditioning therapy and ASCT [16]. In the present study (funded by Dutch Cancer Society, ACTA 2014-7468; trial register NTR5760), 51 MM patients treated with ASCT following HDM (200 mg/m^2^) were included in both Amsterdam University Medical Centre (location Academic Medical Centre (AMC)) or Radboud University Medical Centre (Radboudumc) Nijmegen between September 2015 and March 2017. According to a power analysis using a non-parametric longitudinal data analysis equation (difference in proportion 0.21, *α* of 0.05, 6 sample moments per patient, correlation between follow-up measurements 0.3 and 50% development of severe OM), 48 MM patients needed to be included to ensure a power of 0.80. Patients were excluded when they were not able to understand the provided information, a second SCT was planned in advance, the time before the ASCT was too short to consider study participation or when a transfer to another hospital was planned shortly after ASCT. Ethical approval was obtained (NL52117.018.15) and the study was conducted according to GCP guidelines and the World Medical Association Declaration of Helsinki. Before participating, all patients signed informed consent.

To prevent OM, most patients self-administered cryotherapy during the infusion of HDM. Cryotherapy was started 5–10 min before HDM infusion and was stopped directly after infusion (AMC) or 30 min after infusion (Radboudumc), according to local practice standards. Compliance with the cryotherapy protocol was monitored, but not enforced. Patients treated at the AMC received HDM infused over 2 days (30 min, 100 mg/m^2^ each IV), while patients treated at the Radboudumc received one day infusion of HDM (1 h, 200 mg/m^2^ IV). Safety controls and vital signs monitoring was standard practice including routine laboratory measurements (including blood counts and renal and liver chemistry).

### Saliva collection and OM scoring

Unstimulated and stimulated whole-mouth saliva (UWS and SWS) samples were collected before ASCT during the pre-transplantation dental evaluation (baseline), once a week during the hospitalisation/ASCT phase and 3 and 12 months after ASCT during follow-up dental check-ups. Baseline characteristics were collected during the pre-transplantation dental evaluation. OM was scored using the WHO scoring system 3 times a week during the hospitalisation period (all patients with a score of 2 or above were considered as having ulcerative OM (uOM)) [17]. Before saliva collection patients were asked to refrain from eating and drinking for 1 h. UWS was collected without any stimulation and SWS was collected by chewing on a neutral chewing gum base as previously described [12]. After collection, pH was measured and flow rate was determined using a scale (assumption: 1 ml of saliva is 1 g) [12].

### Salivary analysis

Total protein concentration was determined by absorbance (280 nm, Nanodrop spectrophotometer) for all samples and by bicinchoninic acid assay (BCA) for some of the samples (according to manufacturer’s protocol, Pierce^TM^ BCA protein assay kit, Thermo Scientific, USA). Albumin, lactoferrin, HNP1, secretory IgA (sIgA), S100A8/A9 and total IgA concentrations were determined by enzyme-linked immunosorbent assay (ELISA). Albumin, HNP1 and S100A8/A9 levels were determined in a selection of patients with the highest number and volumes of samples (14 patients of each centre: 7 uOM and 7 non-uOM). For secretory IgA, 3 uOM and 4 non-uOM patients were selected with high total IgA concentrations. All details regarding the ELISAs and the patient characteristics of the subset compared to the entire study population are described in Supplementary File 1.

### Statistical analysis

For the changes in protein concentrations over time, all individual samples were allocated to one of 7 time periods based on day of collection (day 0 is day of ASCT): baseline (base), days −4–0, days 1–5, days 6–11, days 12–18, 3 months (3 M), and 12 months (12 M). Patients were divided into two groups with or without ulcerative OM (uOM and non-uOM). Graphs were made using GraphPad Prism (version 5.03) and statistical analyses were performed in R (version 3.5.1), where *p* < 0.05 was considered statistically significant. Because of non-normal distributions log transformation was applied for the concentrations of total protein, albumin, total IgA, lactoferrin and HNP1. For salivary flow rate, only data from Radboudumc patients were analysed (at the AMC a less sensitive scale was operational). A flow rate of 0 ml/min was recorded if there was no saliva production in 5 min.

Differences between the uOM and non-uOM group was determined at baseline using unpaired *T* tests and for combined time points after baseline using multi-level linear models with random intercepts. Changes in salivary flow rate and pH, and in salivary protein concentrations were first evaluated using graphs. Observed changes over relevant time periods, were subsequently analysed for statistical significance using multi-level regression analysis with random intercepts with OM and time as independent variables. For HNP1 tobit regression was used to analyse outcomes with observations outside detection limits. For the tobit regression, bootstrapping with 1000 replicates was applied to obtain 95% confidence intervals.

## Results

### Study population and saliva collection

In this study, 51 MM patients (53% male) with a median age of 58 years (range 33–69) were included, of which 39% developed uOM (44% AMC/ACTA and 33% Radboudumc) during ASCT phase (Table 1). Peak OM scores were seen between days 6 and 11 in both centres. The patient group from the AMC contained more males, but the melphalan dose in mg/kg and creatinine levels before ASCT were similar in both centres (Table 1).

**Table 1:** Patient characteristics.

	Total (*n* = 51)	AMC (*n* = 27)	Radboudumc (*n* = 24)	uOM (*n* = 20)	Non-uOM (*n* = 31)
Age					
Mean ± SD	57.5 ± 7.3	57.9 ± 8.4	57.0 ± 6.2	58.8 ± 8.6	56.7 ± 6.5
Median	58	62	56	61	56
Range	33–69	33–69	42–69	33–69	42–66
Gender					
Male (%)	27 (52.9)	17 (63.0)	10 (41.7)	10 (50.0)	17 (54.8)
Cryotherapy					
Cryotherapy used (*n* (%))	40 (78.4)	27 (100)	13 (54.2)	17 (85.0)	23 (74.2)
Creatinine levels before ASCT (days −3–0) (µmol/l)					
Mean ± SD	74.6 ± 22.9	73.6 ± 24.9	75.7 ± 21.0	84.7 ± 30.6	68.1 ± 13.1
Median	68	66	70.5	78.5	67
Range	45–145	45–145	49–129	45–145	49–94
Days between start of last chemotherapy cycle and baseline sample					
Mean ± SD	25.2 ± 26.8	23.6 ± 34.5	27.1 ± 14.2	32.6 ± 35.4	20.5 ± 18.5
Median	15	10	19.5	18.5	15
Range	0–114	0–114	9–58	0–114	1–95
Melphalan dose (mg/kg)					
Mean ± SD	5.00 ± 0.45	5.01 ± 0.49	4.98 ± 0.41	4.90 ± 0.40	5.06 ± 0.48
Median	4.96	5.00	4.92	4.86	5.06
Range	4.12–5.87	4.15–5.82	4.12–5.87	4.12–5.61	4.15–5.87
Type M-protein (*n* (%))					
IgA	14 (27.5)	9 (33.3)	5 (20.8)	6 (30.0)	8 (25.8)
IgG	23 (45.1)	12 (44.4)	11 (45.8)	6 (30.0)	17 (54.8)
IgM	1 (2.0)	0	1 (4.2)	0	1 (3.2)
Others	13 (25.5)	6 (22.2)	7 (29.2)	8 (40.0)	5 (16.1)

The baseline saliva samples were collected at a median of 36 days (range −124 to −4) before ASCT. At baseline, patients had already been treated with different chemotherapy cycles (VCD (bortezomib, cyclophosphamide and dexamethasone); cyclophosphamide; or lenalidomide and dexamethasone). The start date of the last chemotherapy cycle was at a median of 15 days before the baseline measurements (range 0–114; Table 1). The 3- and 12 months follow-up visits took place at a median of 105 (range 75–170) and 375 (range 331–506) days after ASCT, respectively.

Although saliva collection was attempted at all time points, some samples were missed during the hospitalisation phase when patients were willing, but unable to provide saliva (due to nausea (9%) or no saliva production (3%); Supplementary File S2). UWS and/or SWS samples were collected at a median of 5 different time points per patient (range 2–6) in both centres.

### Impact of OM

**Fig. 1:**
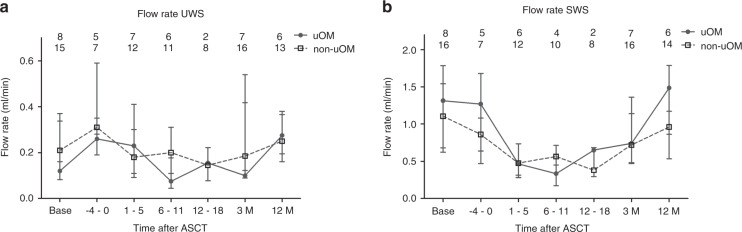
Salivary flow rate over time in uOM and non-uOM groups. Median ± IQR for unstimulated whole-mouth saliva (UWS) (**a**) and stimulated whole-mouth saliva (SWS) (**b**) flow rate over time in the ulcerative oral mucositis (uOM) and non-uOM groups. Numbers in the graphs represent the number of samples at the different time points in the uOM and non-uOM groups.

**Fig. 2:**
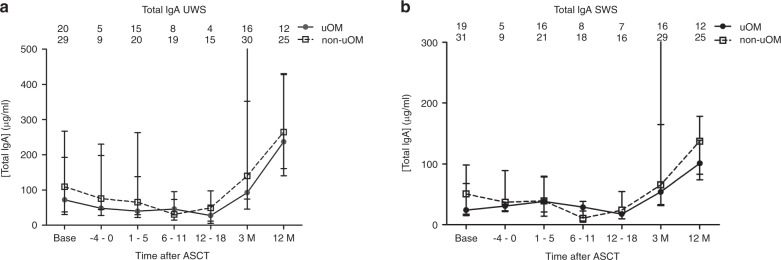
Salivary total IgA concentration over time in uOM and non-uOM groups. Median ± IQR total IgA concentration in unstimulated whole-mouth saliva (UWS) (**a**) and stimulated whole-mouth saliva (SWS) (**b**) over time in the ulcerative oral mucositis (uOM) and non-uOM groups. Numbers in the graph represent the number of samples at the different time points in the uOM and non-uOM groups.

**Fig. 3:**
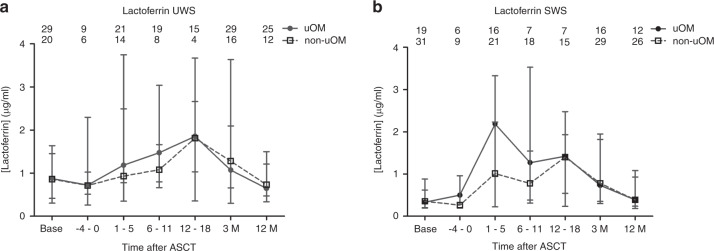
Salivary lactoferrin concentration over time in OM and non-uOM groups. Median ± IQR lactoferrin concentration in unstimulated whole-mouth (UWS) (**a**) and stimulated whole-mouth saliva (SWS) (**b**) over time in the ulcerative oral mucositis (uOM) and non-uOM groups. Numbers in the graph represent the number of samples at the different time points in the uOM and non-uOM groups.

**Fig. 4:**
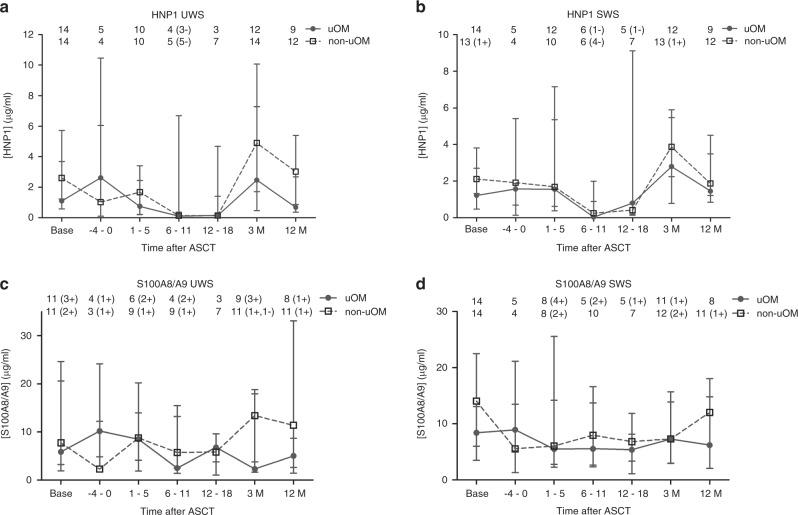
Salivary HNP1 and S100A8/A9 concentration over time in uOM and non-uOM groups. Median ± IQR HNP1 (neutrophil defensin-1) (**a** + **b**) and S100A8/A9 (**c** + **d**) concentration in unstimulated whole-mouth saliva (UWS) (**a** + **c**) and stimulated whole-mouth saliva (SWS) (**b** + **d**) over time in the ulcerative oral mucositis (uOM) and non-uOM groups. Numbers in the graph represent the number of samples at the different time points in the uOM and non-uOM groups. Numbers between brackets represent the amount of samples with readings below (−) or above (+) the detection limit.

Salivary flow rate and concentrations of different salivary proteins were generally not different between the uOM and non-uOM groups (Supplementary File S3, Figs. 1–4).

### Salivary changes over time

SWS flow rate and UWS and SWS pH were significantly decreased up to day 18 compared to baseline levels (see Fig. 1 and Supplementary File S4 for visual presentations and Table 2 for *p* values and 95% CI intervals). Twelve months after ASCT, UWS and SWS flow rate and UWS pH were returned to baseline levels (Table 2). Due to low salivary flow rates and therefore low saliva volumes for some patients, total protein concentrations of all samples were determined using a low volume spectrophotometer (Nanodrop). Although, total protein concentrations were generally higher using Nanodrop, time-dependent trends were similar for Nanodrop and BCA assay (Supplementary File S5). No changes over time were seen for UWS total protein concentrations, while in SWS the graph showed an increase at 1–5 days after ASCT (Supplementary File S5).

**Table 2:** Results of multi-level analysis of pH, flow rate, total IgA, HNP1 and lactoferrin concentration.

	UWS			SWS		
	Effect	95% CI	*p*	Effect	95% CI	*p*
pH (ASCT period)						
Intercept	5.90	[5.83, 5.98]	<0.001	6.27	[6.11, 6.42]	<0.001
OM^a^ (yes = 1, no = 0)	−0.02	[−0.14, 0.11]	0.806	0.10	[−0.15, 0.35]	0.429
Time^b^ (base = 0, 0–18 = 1)	**−0.01**	**[−0.01**, **−0.004]**	**<0.001**	**−0.01**	**[−0.02**, **−0.01]**	**<0.001**
pH (base/12 M)						
Intercept	6.20	[6.09, 6.32]	<0.001	6.94	[6.78, 7.10]	<0.001
OM^a^ (yes = 1, no = 0)	0.02	[−0.13, 0.18]	0.768	0.26	[0.02, 0.49]	0.032
Time^b^ (base = 0, 12 M = 1)	−0.01	[−0.13, 0.11]	0.824	**−0.21**	**[−0.38**, **−0.04]**	**0.013**
Flow rate (ASCT period)						
Intercept	0.23	[0.17, 0.28]	<0.001	0.63	[0.47, 0.78]	<0.001
OM^a^ (yes = 1, no = 0)	−0.06	[−0.15, 0.04]	0.250	0.03	[−0.23, 0.29]	0.830
Time^b^ (base = 0, 0–18 = 1)	−0.001	[−0.002, 0.000]	0.257	**−0.008**	**[−0.01**, **−0.004]**	**<0.001**
Flow rate (base/12 M)						
Intercept	0.29	[0.21, 0.37]	<0.001	1.03	[0.80, 1.26]	<0.001
OM^a^ (yes = 1, no = 0)	−0.08	[−0.21, 0.05]	0.242	0.29	[−0.09, 0.66]	0.137
Time^b^ (base = 0, 12 M = 1)	0.01	[−0.06, 0.07]	0.784	−0.09	[−0.26, 0.08]	0.304
Total IgA^c^ (ASCT period)						
Intercept	4.03	[3.56, 4.50]	<0.001	3.61	[3.12, 4.09]	<0.001
OM^a^ (yes = 1, no = 0)	0.06	[−0.64, 0.76]	0.865	0.04	[−0.70, 0.78]	0.913
Time^b^ (−4–5 = 0, 6–18 = 1)	**−0.52**	**[−0.92**, **−0.12]**	**0.011**	**−0.76**	**[−1.09**, **−0.41]**	**<0.001**
Total IgA^c^ (base/12 M)						
Intercept	5.45	[5.03, 5.86]	<0.001	4.81	[4.40, 5.22]	<0.001
OM^a^ (yes = 1, no = 0)	−0.07	[−0.65, 0.51]	0.815	−0.23	[−0.83, 0.36]	0.435
Time (base = 0, 12 M = 1)	**0.89**	**[0.49**, **1.29]**	**<0.001**	**1.08**	**[0.72**, **1.45]**	**<0.001**
HNP1^c, d^						
Intercept	−0.36	[−1.41, 0.68]^e^	0.496	0.12	[−0.78, 0.91]^e^	0.748
OM^a^ (yes = 1, no = 0)	0.05	[−1.42, 1.68]^e^	0.974	0.49	[−0.63, 1.78]^e^	0.430
Time^b^ (−4–5 = 0, 6–18 = 1)	**−3.42**	**[−5.08**, **−1.91]**^**e**^	**<0.001**	**−3.09**	**[−4.40**, **−1.76]**^**e**^	**<0.001**
Lactoferrin^c^						
Intercept	1.14	[−0.48, 2.75]	0.167	0.16	[−0.94, 1.26]	<0.001
OM^a^ (yes = 1, no = 0)	−0.37	[−2.46, 1.71]	0.727	0.90	[−0.55, 2.35]	0.224
Time^b^ (base = 0, 1–18 = 1)	**2.01**	**[0.36**, **3.58]**	**0.016**	**1.14**	**[1.14**, **3.29]**	**<0.001**

Bold: significant differences over time.

*UWS* unstimulated whole-mouth saliva, *SWS* stimulated whole-mouth saliva, *HNP1* neutrophil defensin-1.

^a^Oral mucositis group.

^b^Time in days.

^c^Log transformed.

^d^Tobit analysis.

^e^Bootstrap analysis.

High variation in albumin concentrations were seen in UWS and SWS with no clear changes over time. Only in the uOM group, the graph showed a small increase at days 6–11 in UWS (Supplementary File S6).

### IgA

Salivary total IgA concentrations were significantly lower in the second and third week after ASCT (days 6–18) compared to the first week (days −4–5), while 12 months after ASCT, total IgA concentrations were higher compared to baseline (see Fig. 2 for visual presentation and Table 2 for *p* values and 95% CI intervals) and highly variable. To determine the salivary gland specific contribution of the total IgA concentration, secretory IgA (sIgA) concentrations were determined for a subset of patients. In general, sIgA concentrations were lower than total IgA concentrations but showed the same time-dependent changes. The very high values for total IgA at baseline, 3 and 12 months after ASCT were not found in the sIgA concentrations (Supplementary File S7).

Excluding the patients with an IgA type MM from the analysis resulted in similar changes over time. Also high levels were still present in the remaining patients, suggesting that the high levels measured were not derived from the MM. To correct for the total IgA levels that may be lost during centrifugation, total IgA concentrations were determined in the pellet fraction. No differences were found between pellet and supernatant total IgA concentrations over time (data not shown).

### Antimicrobial peptides (lactoferrin, HNP1 and S100A8/A9)

A significant increase in lactoferrin concentrations in UWS (effect size 2.01) and SWS (effect size 1.14) was found during the hospitalisation period (see Fig. 3 and Table 2). Twelve months after ASCT, UWS and SWS lactoferrin returned towards baseline concentrations.

Lactoferrin in saliva is derived from the salivary glands and PMNs [12, 18]. To determine the proportion derived from the PMNs, two other salivary antimicrobial peptides derived from PMNs (HNP1 and calprotectin (S100A8/A9)) were studied over time for a subset of the samples. HNP1 concentrations were significantly lower between days 6–18 after ASCT compared to days −4–5 after ASCT in UWS and SWS (effect size −3.42 and −3.09, respectively, see Fig. 4a, b and Table 2). At days 6–11, the HNP1 concentration was below the detection limit in saliva for part of the samples, and while a small increase was seen at 3 months in HNP1 concentration in UWS and SWS, baseline levels were only reached at 12 months (Fig. 4a, b). A similar decreasing trend reaching 0 between days 5 and 11 after ASCT was seen in the number of PMNs in blood in the uOM and non-uOM group, while morning body temperature showed an increasing trend during these days (Supplementary File S8). The S100A8/A9 concentrations fluctuated in UWS and SWS (Fig. 4c, d).

## Discussion

The aim of this study was to determine salivary changes in relation to OM in MM patients treated with HDM followed by ASCT. In the second and third weeks after ASCT, increased lactoferrin concentrations were observed together with decreased concentrations of total IgA, HNP1, pH and diminished UWS and SWS flow rates compared to baseline. Twelve months after ASCT all proteins returned to baseline concentrations, except for total IgA, where concentrations were higher at 12 months after ASCT. No differences were found between the uOM and non-uOM groups with respect to changes in salivary flow rate or salivary protein concentrations.

The decreased concentrations of total IgA, HNP1, pH and diminished UWS and SWS flow rate found in this study, indicate impaired oral mucosal immunity during peak OM scores [11–13]. The decreased total IgA concentration also signifies reduced antimicrobial activity in saliva, possibly compensated to some degree by increased lactoferrin concentrations [12]. There have been indications that glandular inflammation may lead to higher lactoferrin concentrations in saliva. Increased concentrations of lactoferrin were reported during chronic recurrent parotitis and Sjögren’s syndrome [19–22]. In those studies, the tenfold elevation in lactoferrin concentration, which was thus much higher than observed in our study, likely originated from infiltrating PMNs in addition to increased glandular secretion as a result of the inflammatory reaction [19, 21].

In our study, twofold elevated lactoferrin levels were observed during peak OM scores and neutropenia, suggestive of glandular secretion of lactoferrin. HNP1, derived from PMNs granules [12, 18], could also not be detected in saliva during neutropenia and the proportion of lactoferrin derived from the PMNs in the crevicular fluid is therefore expected to be negligible [23]. S100A8/A9, another component of PMNs granules fluctuated over time. This could be due to the inducible release of S100A8/A9 by oral keratinocytes [9, 10]. In lichen planus, candidiasis and other inflammatory diseases of the oral mucosa increased levels of S100A8/A9 were reported [24]. The up-regulation of S100A8/A9 by oral keratinocytes was insufficient to compensate for the considerable reduction in S100A8/A9 levels in UWS and SWS by PMNs during neutropenia.

Elevated lactoferrin might be protective for salivary glands, due to the scavenger and regulatory effects of lactoferrin on cell growth and differentiation as found in irradiated glands [25]. Lactoferrin also inhibits the pro-inflammatory cytokines IL-1, IL-6 and TNF-α [26, 27], which play an important role in the pathogenesis of OM [28]. Lactoferrin has been investigated for use as a preventive agent against infections and inflammatory complications in ASCT patients with unreported success [29, 30]. However, topical administration of bovine lactoferrin on ulcerations in chemotherapy-induced mucositis in hamsters resulted in more and larger ulcerations compared to the control group (treated with bovine serum albumin) [31].

The inflammatory response to HDM is gradually resolved during the hospitalisation period. In an animal model, the cytotoxic effects of 5-fluorouracil in the submandibular and sublingual glands were decreasing 10 days after chemotherapeutic administration [32]. This fits with the return of lactoferrin to baseline levels at 12 months after autologous SCT. The previously reported increase in lactoferrin, β_2_-microglobulin and secretory leucocyte protease inhibitor at 6 months after allogeneic SCT is probably a result of lymphocytic infiltration of the salivary glands due to Graft-versus-Host Disease after allogeneic SCT [11, 14].

Total IgA levels were higher at 12 months after ASCT compared to baseline, but were comparable with the expected sIgA levels in UWS in healthy individuals [33]. This indicates that total IgA levels were already relatively low at baseline, likely as a result of the impaired IgA production by the plasma cells due to the various chemotherapy cycles in the work-up before ASCT [34, 35], with further reduction during the second and third weeks after ASCT due to HDM effects. Increasing levels of total IgA and sIgA at 3 and 12 months after ASCT, reflect the recovery of sIgA production by the salivary glands when the IgA production of the plasma cells is restored [34, 35]. Although the various chemotherapy cycles before baseline also might have induced some damage to the salivary glands resulting in xerostomia and salivary hypofunction [36], total IgA levels were low due to these chemotherapies affecting the number of plasma cells.

**Fig. 5:**
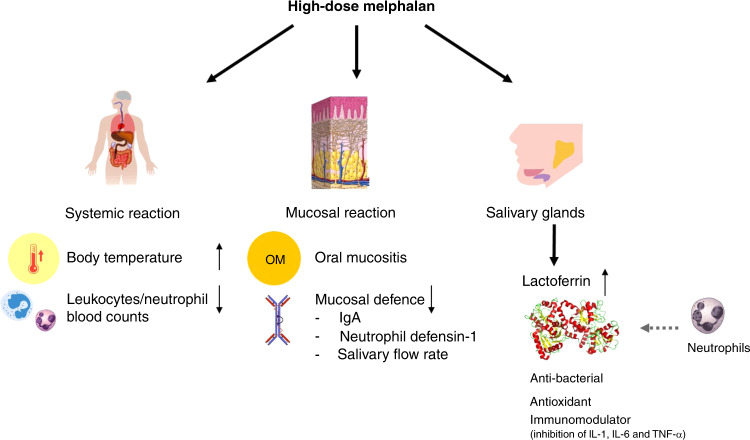
Schematic overview of the hypothesised effects of the inflammatory reactions induced by high-dose melphalan. High-dose melphalan induces a systematic reaction, a mucosal reaction and a reaction in the salivary glands in all patients (uOM and non-uOM). The systemic reaction includes the increase in body temperature and decrease in the leucocytes/neutrophil counts in blood. The mucosal reaction includes oral mucositis and a decreased oral mucosal defence by decreased levels of salivary flow rates, IgA and neutrophil defensin-1 concentrations. Those reactions coincide with the inflammatory reaction in the salivary glands leading to increased lactoferrin concentrations in saliva.

Contrary to expectation, we found no differences between levels of salivary proteins and/or salivary flow rate between uOM and non-uOM patients. We only identified impaired protective functions of saliva by decreased levels of total IgA, HNP1, pH and diminished salivary flow rate and a local glandular inflammatory reaction to HDM by increased lactoferrin concentration. The increased levels of lactoferrin coincided with a systemic inflammatory reaction indicated by neutropenia and increasing body temperature (Fig. 5) [37]. These biological changes were similar between uOM and non-uOM patients, indicating a general inflammatory reaction induced by HDM.

The course of OM found in this study was consistent with a previous report [38]. However, prevalence of uOM was lower, possibly due to cryotherapy [3, 12]. The role of cryotherapy in our study, however, is unclear as the centre with the lowest uOM prevalence also had the lowest cryotherapy compliance (54% vs. 100%). Melphalan dose in mg/kg and creatinine levels in both centres were similar. There was only a small difference in the duration of HDM administration in the low vs. high uOM prevalence centre (1 vs. 2 days) and duration of cryotherapy (stopped 30 min after infusion vs. directly after infusion) that is not of clinical significance.

In conclusion, this study highlights changes in salivary proteins that indicate an inflammatory reaction in the salivary glands coinciding with mucosal and systemic reactions in response to HDM in MM patients, with similar reactions in uOM and non-uOM patients (Fig. 5).

## Supplementary information

Supplemantary file 1

Supplemantary file 2

Supplemantary file 3

Supplemantary file 4

Supplemantary file 5

Supplemantary file 6

Supplemantary file 7

Supplemantary file 8
