# Supplementary material for: Significant salivary changes in relation to oral mucositis following autologous hematopoietic stem cell transplantation
Source: Bone Marrow Transplant. 2021 Jan 8;56(6):1381–90. doi: 10.1038/s41409-020-01185-7 (PMC8189903; doi:10.1038/s41409-020-01185-7)
Supplement: Supplementary file 1 — Supplemantary file 1 [file 41409_2020_1185_MOESM1_ESM.docx]

**Supplementary file 1.** Details ELISA and patients characteristics of the subset used to test albumin, neutrophil defensin-1 and S100A8/A9.

**ELISA details**

For albumin and HNP1, human serum albumin DuoSet ELISA (DY1455, R&D systems) and human alpha-defensin 1 DuoSet ELISA (DY8198-05, R&D systems) kits were used according to manufacturer’s instructions (using half of the recommended volumes per well). Samples were diluted 1:200 and 1:400 for albumin and 1:25 – 1:200 for HNP1. Human S100A8/A9 Heterodimer Quantikine ELISA kit (1:250 and 1:1000 sample dilutions, R&D systems) and Secretory IgA (Human) ELISA kit (1:101 and 1:202 sample dilutions, KA3980, Abnova) were used according to manufacturer’s protocol (3 uOM and 4 non-uOM patients).

For lactoferrin and total IgA, 96 well plates were coated overnight at 4 °C with 50 µl 1:2000 Anti-Lactoferrin antibody (AB15811, Abcam) in PBS + 1% BSA (PBS: 137 mM NaCl, 2.7 mM KCL, 8.1 mM Na_2_HPO_4_ and 1.5 mM KH_2_PO_4_, pH 7.2 – 7.4) or 1:1000 polyclonal rabbit anti-human IgA (A0262 Dako) in 0.1 M Na_2_CO_3_, pH 9.6. For total IgA, plates were washed 3x with PBS-T (0.05% Tween-20 in PBS) and blocked by incubating the plates with 300 µl PBS + 1% BSA for a minimum of 1 hour at RT. Samples were diluted 1:200 and 1:400 in PBS + 1% BSA for lactoferrin and 1:500 and 1:1000 in PBS-T for total IgA. After discarding the first antibody solution or the blocking solution, plates were washed 3x with PBS-T and incubated for 2h at 37 °C with 50 µl sample dilutions or standard (lactoferrin from human milk L0520, Sigma Aldrich (50 ng/ml – 0.8 ng/ml in PBS + 1% BSA) for lactoferrin and IgA from human colostrums I2636, Sigma Aldrich (780 ng/ml – 12.1 ng/ml in PBS-T) for total IgA). Plates were washed 3x with PBS-T and incubated for 1h at 37 °C with 50 µl second antibody (1:25,000 in PBS + 1% BSA Anti-lactoferrin antibody (HRP), AB24264 Abcam; 1:8000 in PBS-T polyclonal rabbit anti-human IgA (HRP), P0216 Dako). After incubation, plates were washed 3x with PBS-T and incubated with 50 µl TMB (TMB liquid substrate system, Sigma Aldrich) at RT in dark and stopped after 20 min with 2 M H_2_SO_4_. Plates were immediately read at 450 and 540 nm using a microplate reader (iMark^TM^ microplate reader Bio-Rad®). Readings at 540 nm were subtracted from 450 nm readings and protein concentrations were calculated using the linear part of the standard curve in Excel (the dilution with readings within the standard curve range was used).

All sample dilutions were tested in duplo. The mean of the calculated concentration of duplo measurements were used in the statistical analysis.

|  | Entire study population (n=51) | Total subset (n=28) | uOM subset (n=14) | non-uOM subset (n=14) |
| --- | --- | --- | --- | --- |
| **Age**  Median  Range | 58  33 – 69 | 56  33 – 69 | 58.5  33 – 69 | 54.5  46 – 66 |
| **Gender** (% male) | 52.9 | 67.9 | 57.1 | 78.6 |
| **Cryotherapy used** (n (%)) | 40 (78.4) | 22 (78.6) | 11 (78.6) | 11 (78.6) |
| **Melphalan dose** (mg/kg)  Mean ± SD  Median  Range | 5.00 ± 0.45  4.96  4.12 – 5.87 | 4.92 ± 0.46  4.82  4.12 – 5.87 | 4.81 ± 0.41  4.81  4.12 – 5.45 | 5.02 ± 0.51  4.98  4.15 – 5.87 |
| **Days between last chemotherapy cycle and baseline sample**  Mean ± SD  Median  Range | 25.2 ± 26.8  15  0 – 114 | 23.3 ± 23.9  15.5  0 – 95 | 24.3 ± 26.2  13  0 – 92 | 22.3 ± 22.3  17.5  5 – 95 |

**Table S1.** Patient characteristics of the subset used to test albumin, neutrophil defensin-1 and S100A8/A9 compared to the entire study population
