# Supplementary material for: Significant salivary changes in relation to oral mucositis following autologous hematopoietic stem cell transplantation
Source: Bone Marrow Transplant. 2021 Jan 8;56(6):1381–90. doi: 10.1038/s41409-020-01185-7 (PMC8189903; doi:10.1038/s41409-020-01185-7)
Supplement: Supplementary file 2 — Supplemantary file 2 [file 41409_2020_1185_MOESM2_ESM.docx]

**Supplementary file 2.** Available samples


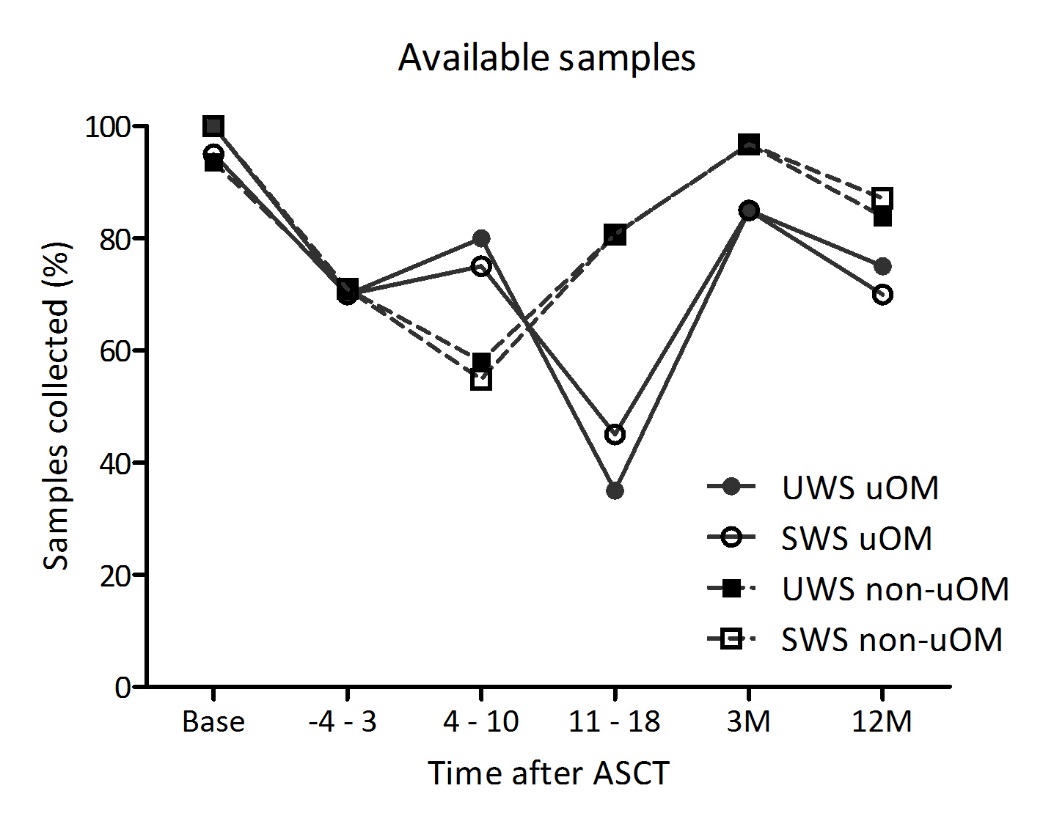


**Figure S1.** Percentage of available samples at the different time points for unstimulated whole-mouth saliva (UWS) and stimulated whole-mouth saliva (SWS) collection in the ulcerative oral mucositis (uOM) and non-uOM groups. Main reasons for missing samples were nausea (overall 9%, 0 – 31%) or no saliva production (overall 3%, 0 – 17%) between 4–18 days after ASCT. Reason for missing samples at 12 months after ASCT was loss of follow-up (disease recurrence or patient refused).
