# Supplementary material for: Significant salivary changes in relation to oral mucositis following autologous hematopoietic stem cell transplantation
Source: Bone Marrow Transplant. 2021 Jan 8;56(6):1381–90. doi: 10.1038/s41409-020-01185-7 (PMC8189903; doi:10.1038/s41409-020-01185-7)
Supplement: Supplementary file 3 — Supplemantary file 3 [file 41409_2020_1185_MOESM3_ESM.docx]

**Supplementary file 3.** Results statistical tests between the ulcerative oral mucositis (uOM) and non-uOM groups at baseline and over all time points (except baseline).

**Table S2.** Results unpaired T-tests for difference between ulcerative oral mucositis (uOM) and non-uOM groups at baseline (1=uOM, 0=non-uOM)

|  | **UWS** | | | | | **SWS** | | |
| --- | --- | --- | --- | --- | --- | --- | --- | --- |
|  | t | 95% CI | | | p | t | 95% CI | P |
| **Flow rate** | 1.67 | | [-0.03, 0.28] | 0.109 | | -0.95 | [-0.70, 0.26] | 0.353 |
| **pH** | 0.13 | | [-0.16, 0.14] | 0.900 | | -0.92 | [-0.48, 0.18] | 0.364 |
| **Total protein^a^** | -0.39 | | [-0.40, 0.27] | 0.700 | | 0.74 | [-0.16, 0.34] | 0.465 |
| **Albumin^a^** | 1.25 | | [-0.63, 2.58] | 0.224 | | -0.11 | [-1.80, 1.62] | 0.915 |
| **Total IgA^a^** | 0.27 | | [-0.66, 0.87] | 0.785 | | 0.87 | [-0.42, 1.07] | 0.390 |
| **Lactoferrin^a^** | -0.10 | | [-0.66, 0.59] | 0.921 | | 0.12 | [-0.47, 0.53] | 0.907 |
| **HNP1^a,b^** | -0.17 | | [-1.33, 0.99] | 0.769 | | -0.57 | [-1.65, 0.51] | 0.297 |
| **S100A8/A9^b^** | 1.89 | | [-12.54, 16.32] | | 0.798 | -3.83 | [-10.76, 3.10] | 0.279 |

^a^Log transformation, ^b^Tobit regression

**Table S3.** Results of linear multi-level analysis for difference between ulcerative oral mucositis (uOM) and non-uOM groups over all time points, without baseline (1=uOM, 0=non-uOM).

|  | **UWS** | | | **SWS** | | |
| --- | --- | --- | --- | --- | --- | --- |
|  | Effect | 95% CI | p | Effect | 95% CI | P |
| **Flow rate** | -0.002 | [-0.11, 0.10] | 0.964 | -0.156 | [-0.40, 0.09] | 0.211 |
| **pH** | -0.027 | [-0.17, 0.12] | 0.714 | -0.146 | [-0.37, 0.08] | 0.201 |
| **Total protein^a^** | -0.086 | [-0.31, 0.14] | 0.440 | -0.188 | [-0.40, 0.02] | 0.079 |
| **Albumin^a^** | 0.235 | [-0.83, 1.30] | 0.663 | 0.011 | [-1.15, 1.17] | 0.987 |
| **Total IgA^a^** | -0.060 | [-0.62, 0.49] | 0.827 | -0.024 | [-0.60, 0.55] | 0.939 |
| **Lactoferrin^a^** | -0.326 | [-0.90, 0.24] | 0.260 | -0.320 | [-0.87, 0.23] | 0.255 |
| **HNP1^a,b^** | 0.113 | [-1.17, 1.27] | 0.856 | 0.208 | [-0.67, 1.04] | 0.636 |
| **S100A8/A9^a,b^** | 2.24 | [-6.55, 12.15] | 0.664 | 3.680 | [-3.57, 10.66] | 0.294 |

^a^Log transformation, ^b^Bootstrap of Tobit regression
