# Supplementary material for: Significant salivary changes in relation to oral mucositis following autologous hematopoietic stem cell transplantation
Source: Bone Marrow Transplant. 2021 Jan 8;56(6):1381–90. doi: 10.1038/s41409-020-01185-7 (PMC8189903; doi:10.1038/s41409-020-01185-7)
Supplement: Supplementary file 4 — Supplemantary file 4 [file 41409_2020_1185_MOESM4_ESM.docx]

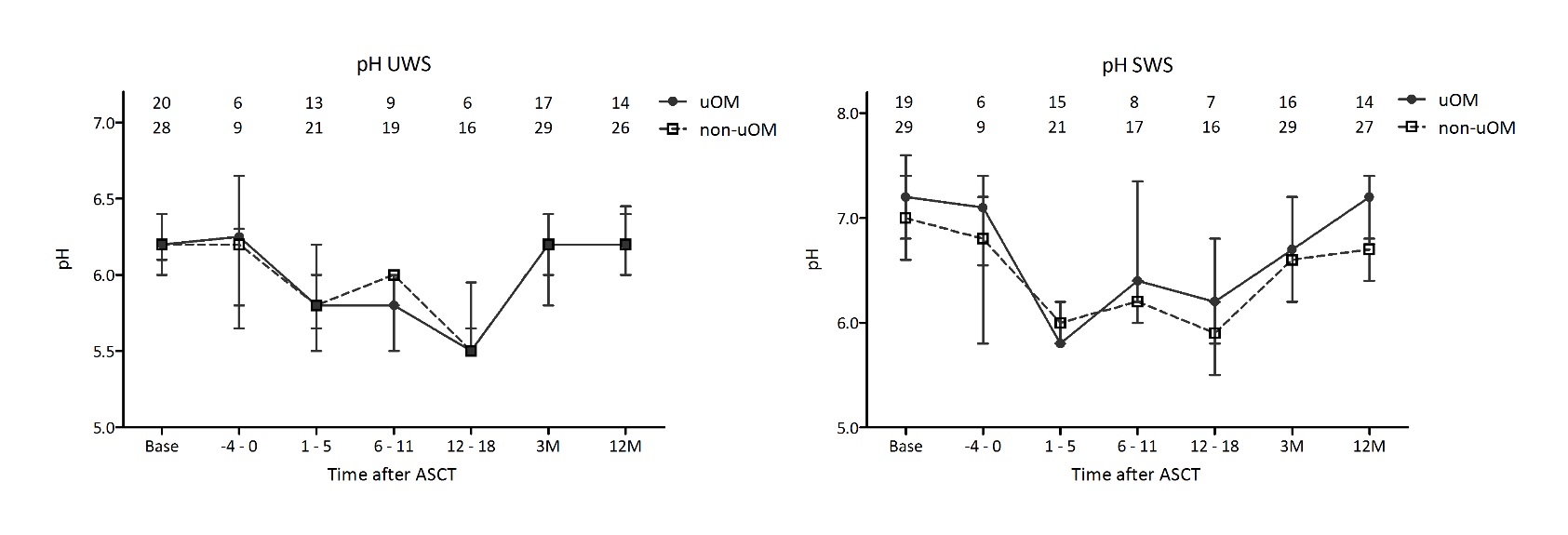
**Supplementary file 4.** pH in UWS and SWS

**Figure S2.** Median ± IQR for unstimulated whole-mouth saliva (UWS) and stimulated whole-mouth saliva (SWS) pH over time in the ulcerative oral mucositis (uOM) and non-uOM groups. Numbers in the graphs represent the number of samples at the different time points in the uOM and non-uOM groups.
