# Supplementary material for: Significant salivary changes in relation to oral mucositis following autologous hematopoietic stem cell transplantation
Source: Bone Marrow Transplant. 2021 Jan 8;56(6):1381–90. doi: 10.1038/s41409-020-01185-7 (PMC8189903; doi:10.1038/s41409-020-01185-7)
Supplement: Supplementary file 5 — Supplemantary file 5 [file 41409_2020_1185_MOESM5_ESM.docx]

**Supplementary file 5.** Total protein


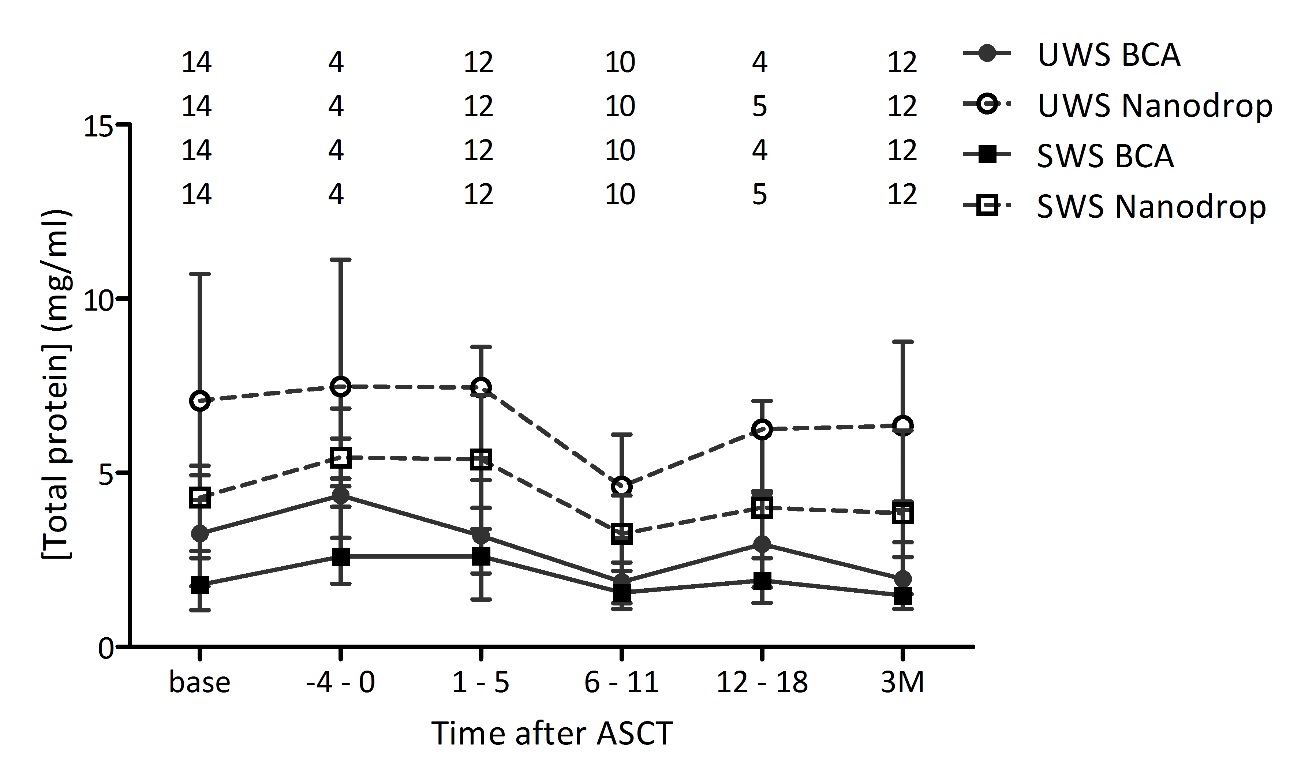


**
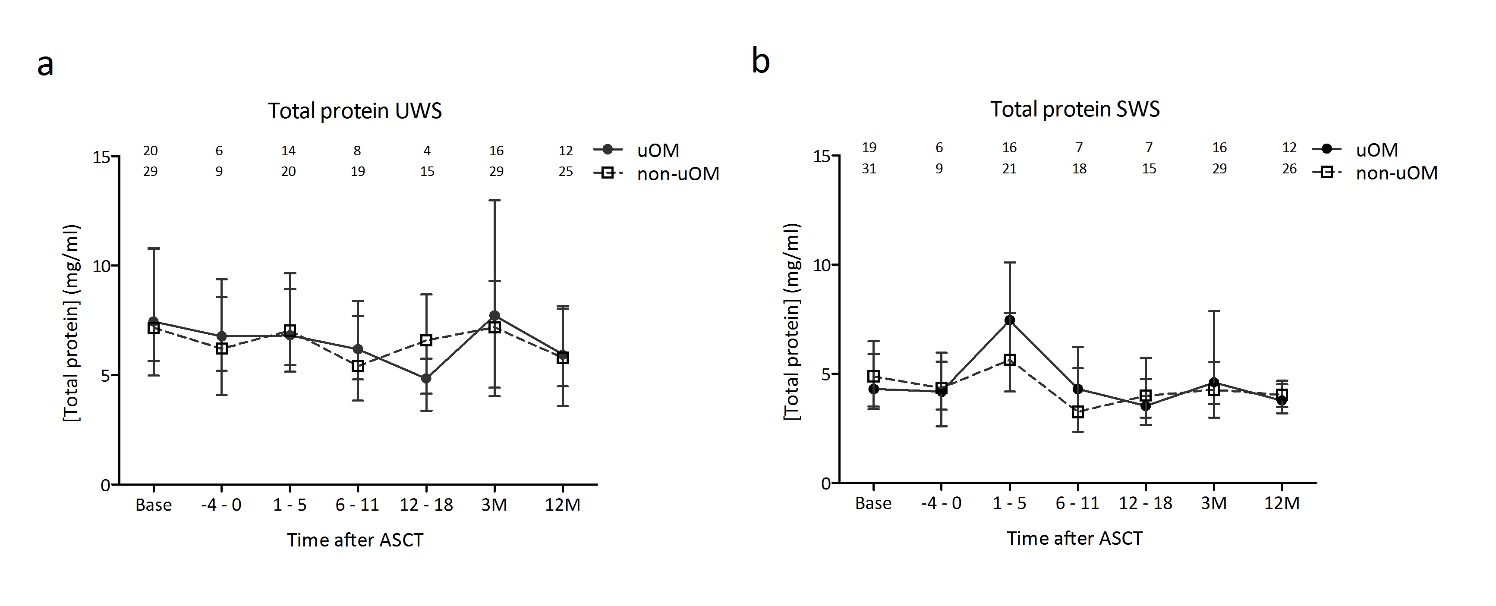
Figure S3.** Unstimulated whole-mouth saliva (UWS) and stimulated whole-mouth saliva (SWS) total protein concentrations (median ± IQR) over time measured with Nanodrop and BCA assay. Numbers in the graph represent the number of samples at the different time points.

**Figure S4.** Median ± IQR total protein concentration in unstimulated whole-mouth saliva (UWS) (a) and stimulated whole-mouth saliva (SWS) (b) over time in the ulcerative oral mucositis (uOM) and non-uOM groups. Numbers in the graphs represent the number of samples at the different time points in the uOM and non-uOM groups.
