# Supplementary material for: Significant salivary changes in relation to oral mucositis following autologous hematopoietic stem cell transplantation
Source: Bone Marrow Transplant. 2021 Jan 8;56(6):1381–90. doi: 10.1038/s41409-020-01185-7 (PMC8189903; doi:10.1038/s41409-020-01185-7)
Supplement: Supplementary file 6 — Supplemantary file 6 [file 41409_2020_1185_MOESM6_ESM.docx]

**Supplementary file 6.** Albumin concentration in UWS and SWS


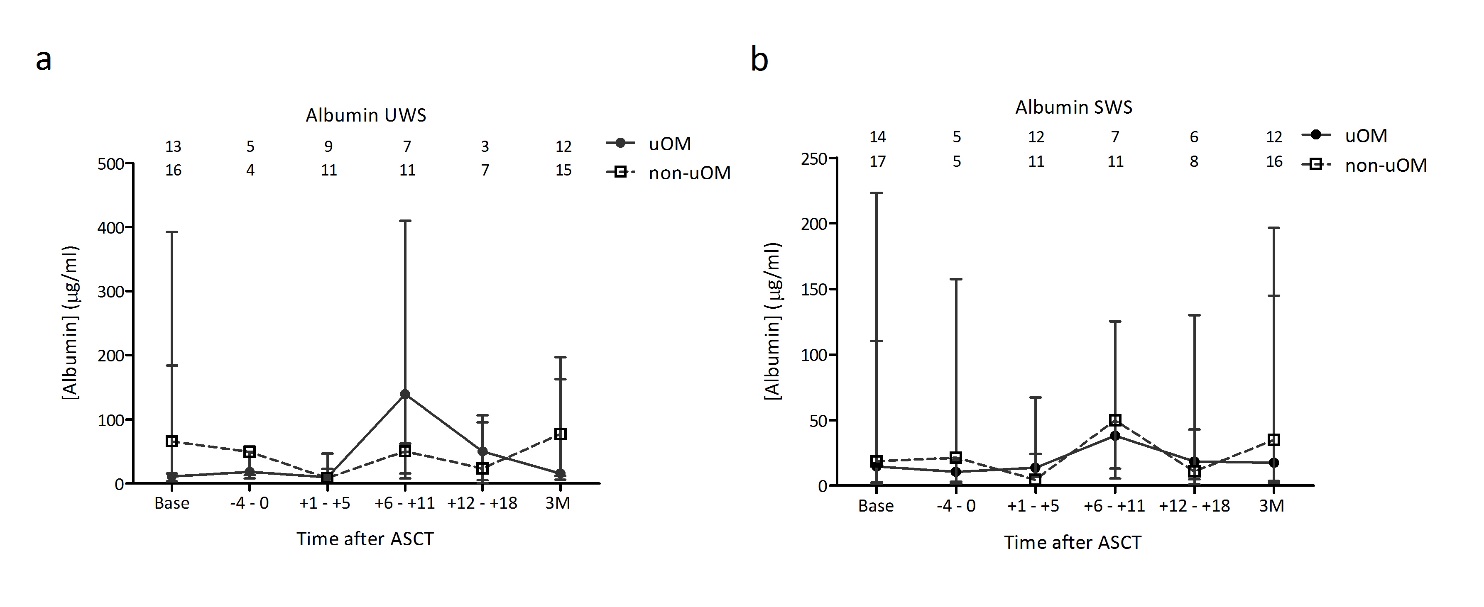


**Figure S5.** Median ± IQR albumin concentration in unstimulated whole-mouth saliva (UWS) (a) and stimulated whole-mouth saliva (SWS) (b) over time in the ulcerative oral mucositis (uOM) and non-uOM groups. Numbers in the graph represent the number of samples at the different time points in the uOM and non-uOM groups.
