# Supplementary material for: Significant salivary changes in relation to oral mucositis following autologous hematopoietic stem cell transplantation
Source: Bone Marrow Transplant. 2021 Jan 8;56(6):1381–90. doi: 10.1038/s41409-020-01185-7 (PMC8189903; doi:10.1038/s41409-020-01185-7)
Supplement: Supplementary file 7 — Supplemantary file 7 [file 41409_2020_1185_MOESM7_ESM.docx]

**Supplementary file 7.** Total IgA and secretory IgA (sIgA)


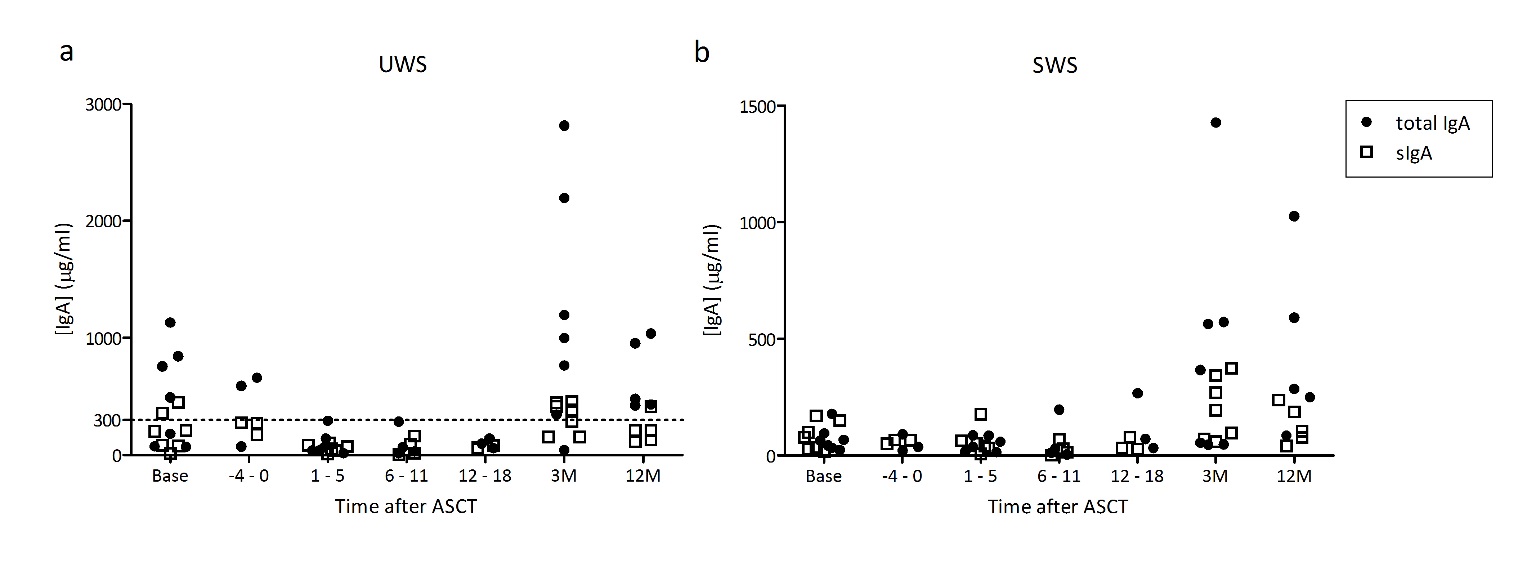


**Figure S6.** Scatterplot of total IgA (black dots) and sIgA (open squares) levels in unstimulated whole-mouth saliva (UWS) (a) and stimulated whole-mouth saliva (SWS) (b) over time. The dotted line in graph (a) represents the expected sIgA level in healthy individuals, based the findings of Prodan *et al.* 2015 (33).
