# Supplementary material for: Significant salivary changes in relation to oral mucositis following autologous hematopoietic stem cell transplantation
Source: Bone Marrow Transplant. 2021 Jan 8;56(6):1381–90. doi: 10.1038/s41409-020-01185-7 (PMC8189903; doi:10.1038/s41409-020-01185-7)
Supplement: Supplementary file 8 — Supplemantary file 8 [file 41409_2020_1185_MOESM8_ESM.docx]

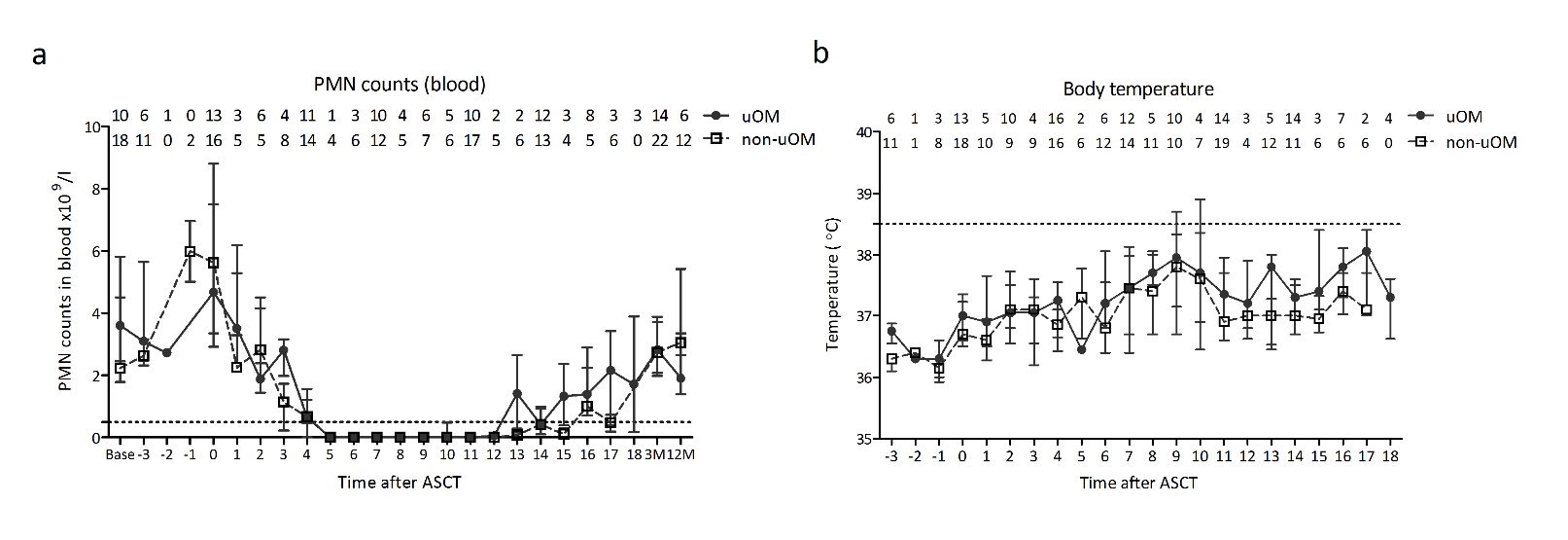
**Supplementary file 8.** PMN counts and body temperature

**Figure S7.** Median ± IQR PMN counts in blood (a) and morning body temperature (b) over time in the ulcerative oral mucositis (uOM) and non-uOM groups. Numbers in the graph represent the number of patients at the different time points in the uOM and non-uOM groups. Lines in graphs represent thresholds for neutropenia (a) and fever (b), respectively.
